# Supplementary material for: Genome-Wide Comparative Analysis of the R2R3-MYB Gene Family in Five Solanaceae Species and Identification of Members Regulating Carotenoid Biosynthesis in Wolfberry
Source: Int J Mol Sci. 2022 Feb 18;23(4):2259. doi: 10.3390/ijms23042259 (PMC8875911; doi:10.3390/ijms23042259)
Supplement: Supplementary file 1 [file ijms-23-02259-s001.zip › Supplementary/Figure Supplementary/Figure S1 Sequence conservation of the conserved domain of R2R3-MYB proteins among five Solanaceae specie.pdf]

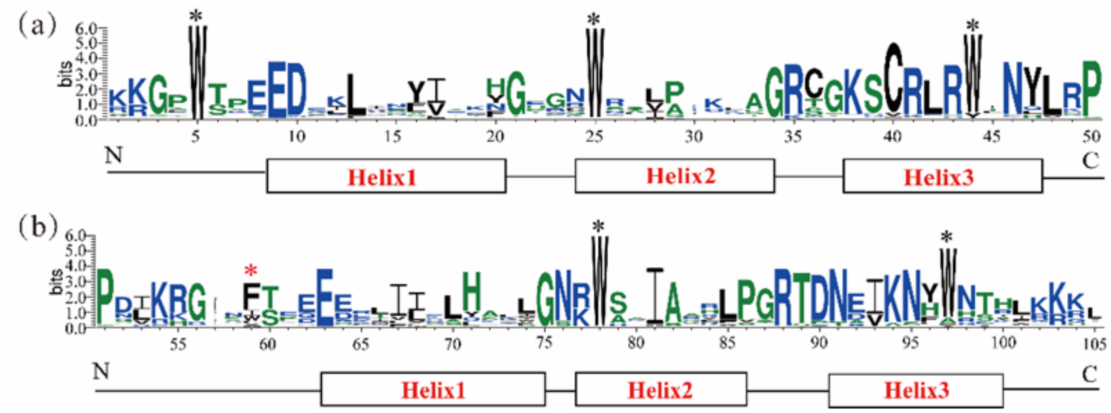

Figure.S1 Sequence of logo of R2 (a) and R3 (b) MYB domain are based on the full-length alignments of all R2R3-MYB domains in five Solanaceae species: *Lycium barbarum*, *Solanum lycopersicum*, *Capsicum annuum*, *Solanum tuberosum* and *Solanum melongena*. The bit score exhibits the information content for each position in the sequence. The position of the three-helices is marked (Helix 1 to 3). Black asterisk indicates the position of the conserved amino acid that are identical among R2R3-MYB proteins. The replaced residues in the R3 domain repeat are shown by red asterisks.
