# Supplementary material for: Genome-Wide Comparative Analysis of the R2R3-MYB Gene Family in Five Solanaceae Species and Identification of Members Regulating Carotenoid Biosynthesis in Wolfberry
Source: Int J Mol Sci. 2022 Feb 18;23(4):2259. doi: 10.3390/ijms23042259 (PMC8875911; doi:10.3390/ijms23042259)
Supplement: Supplementary file 1 [file ijms-23-02259-s001.zip › Supplementary/Figure Supplementary/Figure S4 Relationship between the number of R2R3-MYB genes and Chromosome length.pdf]

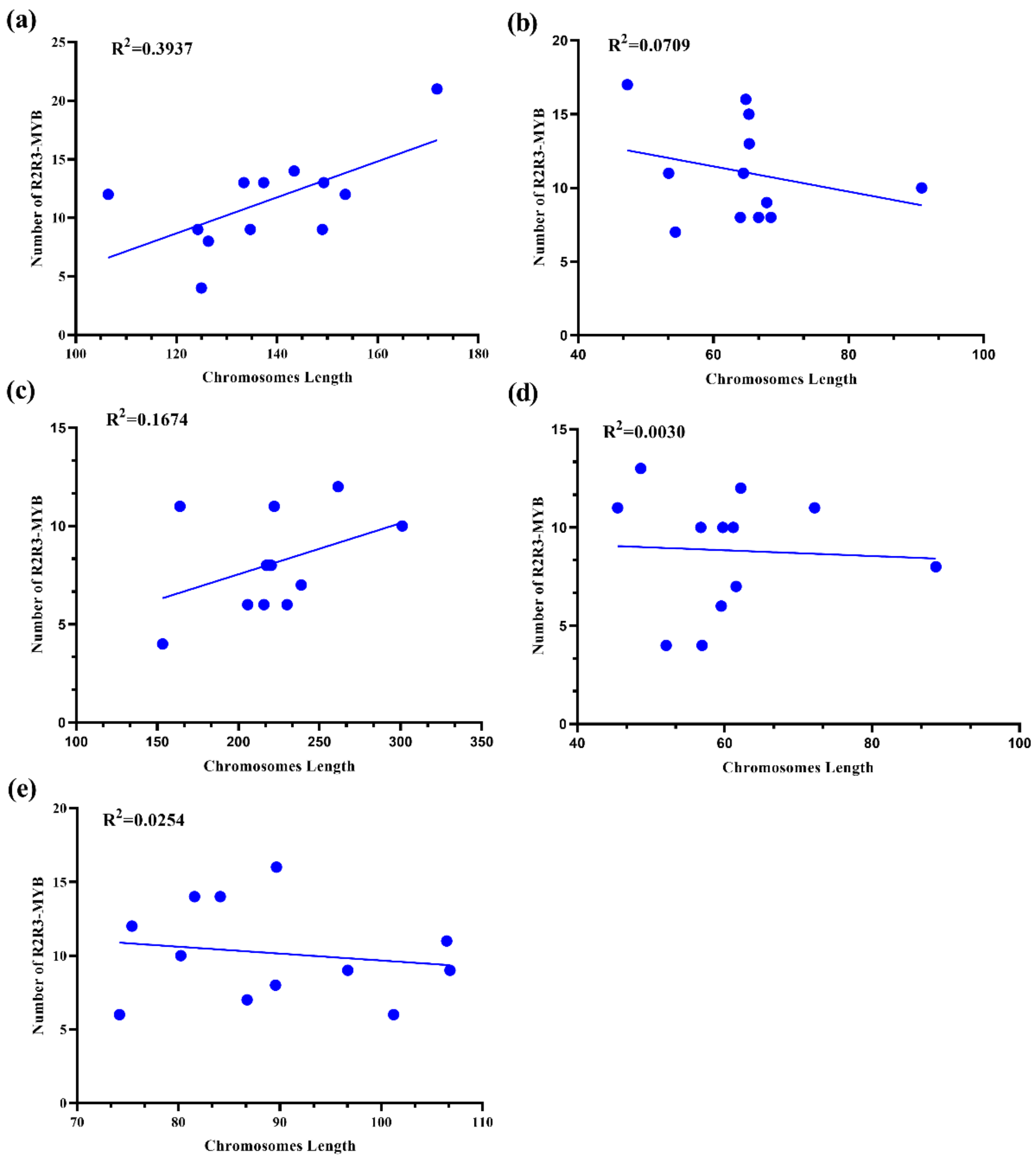

Figure S4. Relationship between the number of *R2R3-MYB* genes and Chromosome length. The x-axis represents chromosomes length. The y-axis represents the number of *R2R3-MYB* genes. (a) wolfberry, (b) tomato, (c) pepper, (d) potato, (e) eggplant
