# Supplementary material for: Genome-Wide Comparative Analysis of the R2R3-MYB Gene Family in Five Solanaceae Species and Identification of Members Regulating Carotenoid Biosynthesis in Wolfberry
Source: Int J Mol Sci. 2022 Feb 18;23(4):2259. doi: 10.3390/ijms23042259 (PMC8875911; doi:10.3390/ijms23042259)
Supplement: Supplementary file 1 [file ijms-23-02259-s001.zip › Supplementary/Figure Supplementary/Figure S5 Ks value of five Solanaceae species.pdf]

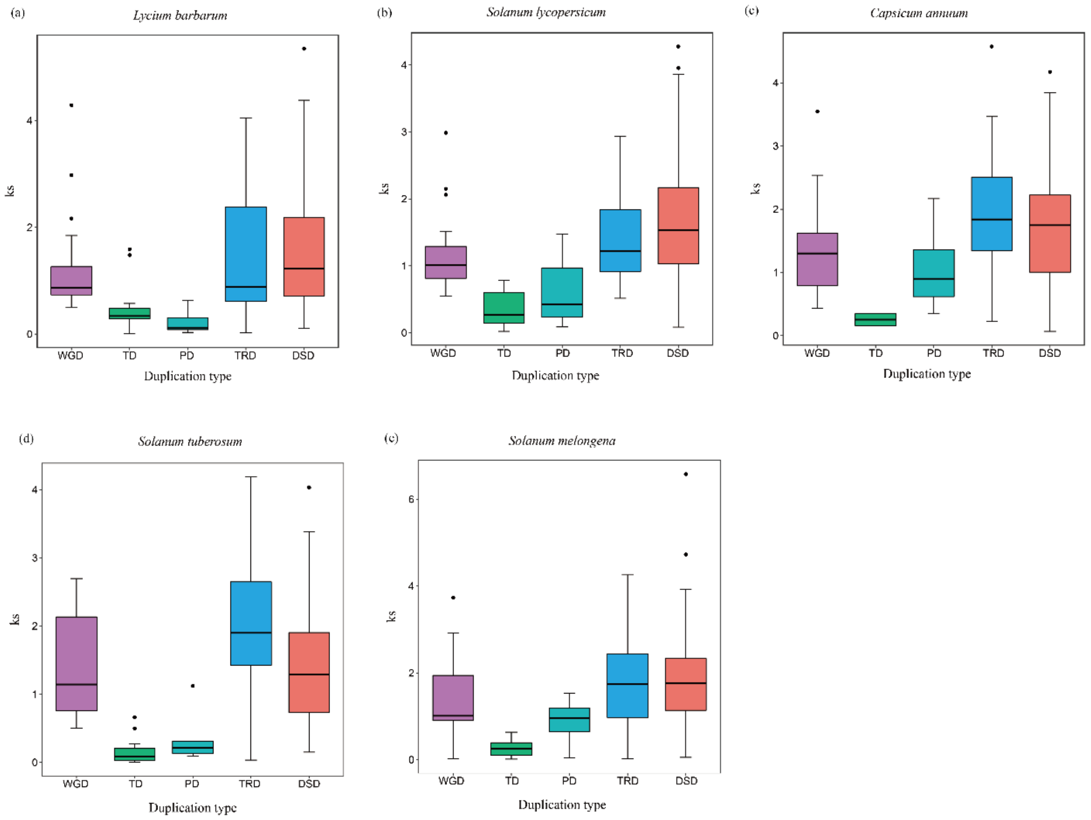

Figure S5. Ks value of five Solanaceae species. The *x-axis* represents five different duplication type. WGD: whole-genome duplicates; TD: tandem duplicates; PD: proximal duplicates; TRD: transposed duplicates; DSD: dispersed duplicated. The *y-axis* indicates the Ka/Ks ratio. (a): wolfberry; (b): tomato; (c): pepper; (d): potato, (e): eggplant
