# Supplementary figures and images for: Genome-Wide Comparative Analysis of the R2R3-MYB Gene Family in Five Solanaceae Species and Identification of Members Regulating Carotenoid Biosynthesis in Wolfberry
Source: Int J Mol Sci. 2022 Feb 18;23(4):2259. doi: 10.3390/ijms23042259 (PMC8875911; doi:10.3390/ijms23042259)

**bootstrap**

- 50
- 62.5
- 75
- 87.5
- 100

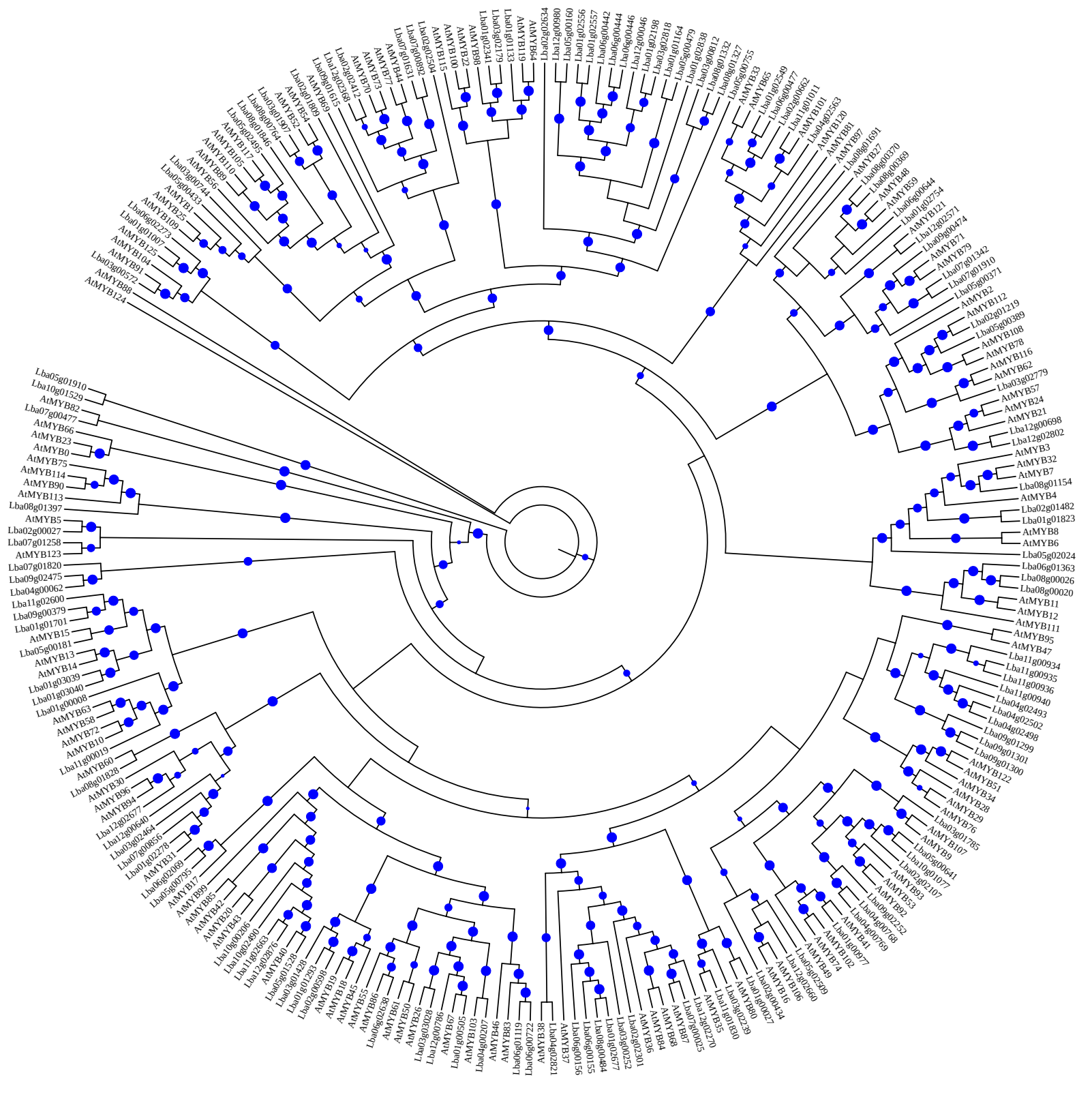

Supplement: Supplementary file 1 [file ijms-23-02259-s001.zip › Supplementary/Figure Supplementary/Figure S2 Phylogenetic analysis of R2R3-MYB proteins between Wolfberry and Arabidopsis.pdf]

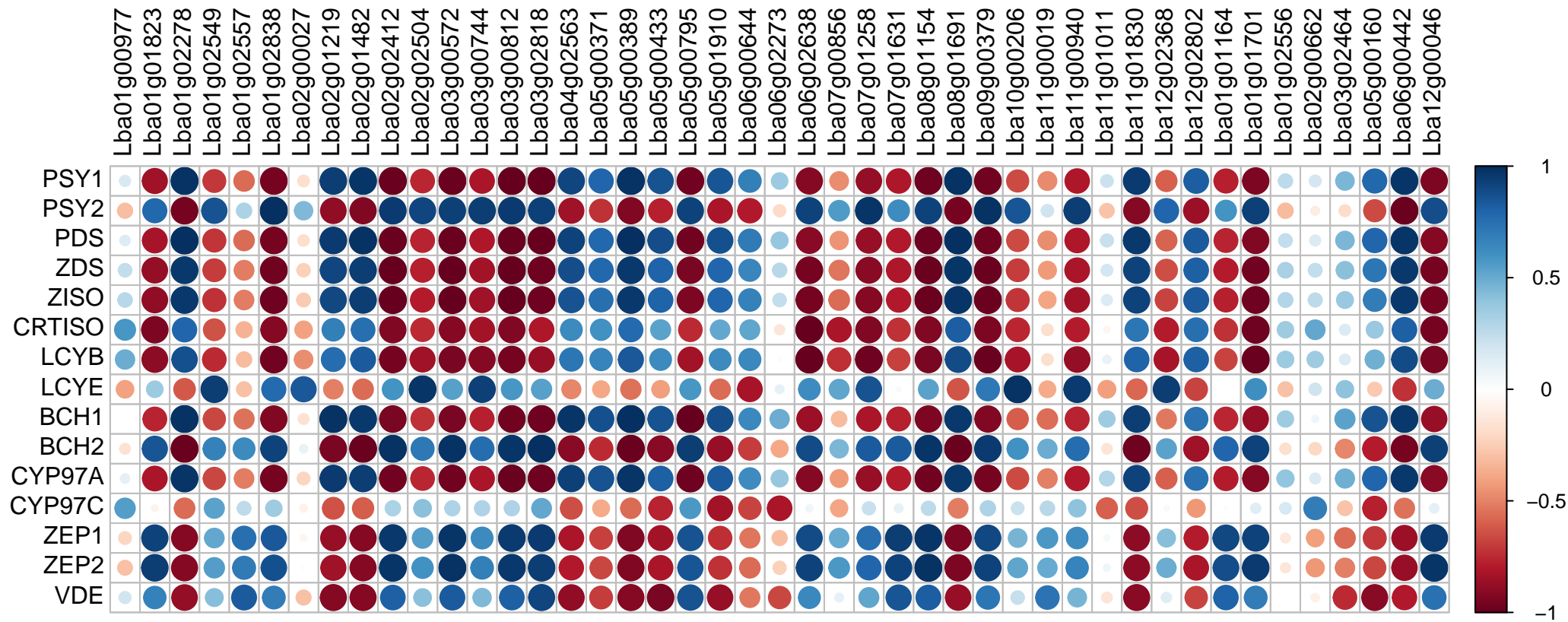

Supplement: Supplementary file 1 [file ijms-23-02259-s001.zip › Supplementary/Figure Supplementary/Figure S6 Pearson's correlation coefficient between LbaR2R-MYB genes and carotenoids biosynthesis genes .pdf]
