# Supplementary material for: Genome-Wide Comparative Analysis of the R2R3-MYB Gene Family in Five Solanaceae Species and Identification of Members Regulating Carotenoid Biosynthesis in Wolfberry
Source: Int J Mol Sci. 2022 Feb 18;23(4):2259. doi: 10.3390/ijms23042259 (PMC8875911; doi:10.3390/ijms23042259)

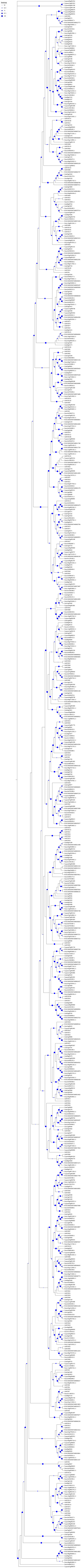

Supplement: Supplementary file 1 [file Figure S3 Phylogenetic analysis of the R2R3-MYB proteins of wolfberry, tomato, pepper, tomato, eggplant and Arabidopsis.pdf › Supplementary/Figure Supplementary/Figure S3 Phylogenetic analysis of the R2R3-MYB proteins of wolfberry, tomato, pepper, tomato, eggplant and Arabidopsis.pdf]
